# Assessment of Common Oral Behaviors in Patients with Temporomandibular Joint Disorders and Their Relationship to Psychosocial Factors

**DOI:** 10.3390/dj13100480

**Published:** 2025-10-20

**Authors:** Nguyen Ngoc Hoa, Hoang Viet Hai, Tran Thai Binh, To Thanh Dong, Tran Thi Minh Quyen, Toan Do

**Affiliations:** 1 School of Dentistry, Hanoi Medical University, 1 Ton That Tung Street, Dong Da, Hanoi 100000, Vietnam; nguyenngochoa@hmu.edu.vn (N.N.H.); hoangviethai@hmu.edu.vn (H.V.H.); 2 108 Military Central Hospital, 1B Tran Hung Dao Street, Hai Ba Trung, Hanoi 100000, Vietnam; dr.tranthaibinh@gmail.com; 3 Department of Mathematics and Informatics, Hanoi Medical University, 1 Ton That Tung Street, Dong Da, Hanoi 100000, Vietnam; tothanhdong@hmu.edu.vn; 4 Faculty of Traditional Medicine, Hanoi Medical University, 1 Ton That Tung Street, Dong Da, Hanoi 100000, Vietnam; minhquyendr@hmu.edu.vn; 5 Department of Research Methodology and Biostatistics, School of Preventive Medicine and Public Health, Hanoi Medical University, 1 Ton That Tung Street, Dong Da, Hanoi 100000, Vietnam

**Keywords:** temporomandibular joint disorders, oral behaviors, psychological factors, psychological characteristics, psychological disorders

## Abstract

**Background:** Temporomandibular disorders (TMDs) exhibit a complex relationship with depression, anxiety disorders, and oral behaviors. This cross-sectional study aimed to assess the differences in oral behaviors among subgroups of TMD-related pain and patients with varying levels of anxiety and depression. **Methods:** A total of 120 patients diagnosed with TMD according to the Diagnostic Criteria for Temporomandibular Disorders (DC/TMD), completed the Oral Behavior Checklist (OBC), Visual Analog Scale (VAS), Patient Health Questionnaire-9 (PHQ-9), General Anxiety Disorder (GAD-7), Graded Chronic Pain Scale (GCPS), Patient Health Questionnaire-15 (PHQ-15), and Jaw Functional Limitation Scale-8 (JFLS-8). Associations were examined using Chi-square, Fisher’s exact, and Spearman’s correlation; logistic regression and multivariable linear regression were performed (*p* < 0.05). **Results:** In univariate analyses, several item-level OBs were more prevalent with higher anxiety, including nocturnal bruxism, sleep positions exerting jaw pressure, daytime grinding, and gum chewing (*p* = 0.007, 0.041, 0.011, and 0.014, respectively). A modest difference among pain subgroups was observed for sleep position (*p* = 0.044). In multivariable models, anxiety was independently associated only with nocturnal bruxism (OR = 2.95; 95% CI: 1.30–6.67; *p* = 0.010), whereas depression showed no independent associations. Pain intensity remained the sole predictor of total OBC scores (Coef = 1.829; 95% CI: 0.51–3.15; *p* = 0.007). No independent effects were detected for TMD subgroups. **Conclusions:** Psychosocial factors appeared related to OBs in univariate analyses, but these associations were explained by confounding influences. After adjustment, pain intensity and anxiety emerged as key determinants. These findings highlight the need for comprehensive TMD management integrating pain control with behavioral strategies.

## 1. Introduction

Temporomandibular joint disorders are a prevalent etiology of orofacial pain, exhibiting a rising incidence in clinical practice [1]. The overall prevalence of Temporomandibular Joint Disorders was approximately 31% for adults and 11% for adolescents [2]. The prevalence of TMDs was significantly higher in South America (47%) compared to Asia (33%) and Europe (29%) [3]. In South Korea, a study analyzing 2017 health insurance claims data reported that TMD patients visited healthcare facilities an average of 2.4 times yearly. The total cost was 721,447.5 US$ on inpatient care and the rest on outpatient care. The cost of inpatient care was 7.4 times higher than that of outpatient care, at an average of 71.4 and 527.8 US$ for outpatients and inpatients, respectively [4]. This is a multifactorial disease group involving malocclusion, craniofacial trauma, as well as neuropsychological and oral behavioral elements [5]. Psychological and social factors interact with pain, influencing its perception, intensity, duration, and treatment response. However, they are typically not the only contributing factors [6].

The Diagnostic Criteria for Temporomandibular Disorders (DC/TMD) employs a biopsychosocial model, integrating general health, pain-related symptoms, bruxism, and psychological, social, and genetic factors [7]. The DC/TMD guidelines recommend using the Oral Behavior Checklist (OBC) as a reliable screening tool for identifying harmful oral habits. Widely validated and employed in research, the OBC enables comprehensive assessments of oral behaviors contributing to TMD onset and progression [8]. Oral behaviors are defined as abnormal oral activities that deviate from normal physiological functions, including gum chewing, bruxism, nail biting, and lip biting [9]. These habits can lead to masticatory muscle dysfunction, tissue damage around the temporomandibular joint, pain, and functional limitations [10]. Oral behaviors contribute to microtrauma, particularly in the temporomandibular joint [1]. Current research has demonstrated that non-physiological oral behaviors are implicated in the etiology of TMD pain and may act as significant risk factors. Persistent, even mild, oral habits can contribute to the progressive degeneration of the temporomandibular joint [11]. Oral behaviors are strongly correlated with the presence of TMD pain. Individuals with a higher frequency of oral behaviors are at increased risk of experiencing TMD symptoms [12].

TMDs are frequently associated with symptoms of anxiety, depression, and physical symptoms, even when these conditions are not severe [13]. A complex, bidirectional relationship exists among TMDs, stress, depression, anxiety, and oral behaviors, as evidenced by research data. Individuals with TMDs report higher rates of stress, depression, and anxiety compared to a healthy control group [14]. High levels of oral behaviors and OBC scores are associated with chronic TMD pain [15]. Prolonged chronic pain can lead to an increased likelihood of developing depression [16]. Depression and anxiety also increase the risk of developing TMDs. Individuals with depressive symptoms are more likely to exhibit oral behavioral disorders [13,17]. Alternatively, oral behaviors have been suggested as both avoidance mechanisms and indicators of psychological stress. While certain behaviors like bruxism and clenching have been implicated as risk factors for TMDs, not all studies agreed with this viewpoint [8,12,18]. Some studies have failed to find a significant association between abnormal oral habits and facial pain [19]. There is only a weak association between anxiety and the frequency of oral behaviors in pain-free individuals [8].

While parafunctional habits are widely recognized as significant risk factors for temporomandibular joint disorders, the precise nature of the causal relationship remains elusive [20]. Several studies have explored the association between specific TMD subgroups and psychosocial factors, including oral habits, depression, and anxiety [12,14,17]. However, inconsistencies in subgroup definitions across studies have hindered direct comparisons [14,15,17]. We hypothesized that anxiety levels and depression are significantly associated with oral behaviors in patients with TMDs. This study aims to assess the differences in oral behaviors among various TMD subgroups and explore their association with varying levels of anxiety and depression.

## 2. Methods

### 2.1. Study Design

A cross-sectional design was used for this study.

### 2.2. Study Setting

A cohort of 120 patients diagnosed with TMDs based on the DC/TMD criteria was recruited from the practice facilities of the School of Dentistry, Hanoi Medical University, Vietnam, during the study period from April 2023 to July 2024. This is one of the largest healthcare centers and a leading facility in the management of temporomandibular joint disorders in Vietnam, with a wide variety of cases and a large number of patients. It serves both urban and rural populations across northern Vietnam, thus ensuring a diverse and representative clinical sample. However, as this was a hospital-based population, findings may not be fully generalizable to the broader community.

### 2.3. Participants

Inclusion criteria: Patients aged 16 years and older who can comprehend medical instructions and adhere to examination protocols.

Exclusion criteria: A history of severe neurological disorders, autoimmune diseases affecting the joints and muscles, advanced malignancies, psychiatric conditions, substance abuse (including alcohol, drugs, and analgesics), facial or head trauma (especially fractures within the past 10 years), rheumatoid arthritis, psoriatic arthritis, gout, or any systemic diseases that could potentially impact the masticatory system or cause facial or jaw inflammation [17]. Patients with a history of neck and facial surgery or radiation therapy within the past 3 months or those who have received other treatments for neck or temporomandibular joint conditions were excluded. Additionally, patients taking medications known to affect the neuromuscular system and pregnant women were not included in the study [21].

### 2.4. Variables and Data Measurement

All patients self-administered the questionnaire. The Oral Behavior Checklist (OBC) is a standardized and internationally validated questionnaire designed to assess parafunctional behaviors, recommended by the DC/TMD Axis II instruments [22,23]. There are 21 oral behaviors listed in the OBC, including: clench or grind teeth when asleep; sleep in a position that puts pressure on the jaw; grind teeth together during walking hours; use chewing gum; chew food on one side only; sustained talking; yawning; other oral behaviors (clench teeth together during walking hours; press, touch or hold teeth together other than while eating; hold, tighten or tense muscles without clenching or bringing teeth together; hold or jut jaw forward or to the side; press tongue forcibly against teeth; place tongue between teeth; bite, chew or play with tongue, cheeks or lips, hold jaw in rigid or tense position; hold between the teeth or bite objects; play musical instrument that involves use of mouth or jaw; lean with your hand on the jaw; eating between meals; singing; hold telephone between head and shoulders) [22]. It assigns a score based on the sum of responses, with each item rated on a scale of 0 to 4. Comparisons between individuals with and without chronic temporomandibular disorders (TMDs) have shown that scores between 0 and 16 are considered normal. However, scores between 17 and 24 are twice as prevalent in individuals with TMDs, and scores of 25 or higher are associated with a significantly increased risk of developing TMDs [23].

Other psychosocial factors such as GCPS 2.0, JFLS-8, PHQ-9, GAD-7, and PHQ-15 were assessed using internally validated DC/TMD Axis II questionnaires [23,24,25,26]. The GCPS 2.0, a tool specifically designed to assess chronic pain, consists of eight items that measure both pain intensity and functional limitations. Classification by level is as follows: Grade 0 no pain; Grade I mild dysfunction with low pain intensity; Grade II mild dysfunction with high pain intensity; Grade III moderate dysfunction with moderate limitations; Grade IV severe dysfunction with severe restrictions [24,25]. The JFLS-8 assesses the degree of mandibular dysfunction during activities such as chewing tough food, chewing chicken, eating soft food requiring no chewing, opening wide enough to drink from a cup, swallowing, yawning, talking, and smiling. If a patient completely avoids an activity due to difficulty, they will circle ‘10’. If a patient avoids an activity for reasons unrelated to pain or difficulty, they will leave that item blank [23,26]. PHQ-9 was administered to assess depressive symptoms based on the DC/TMD criteria [23,26]. The scoring is as follows: not at all: 0 points; several days: 1 point; more than half the days: 2 points; nearly every day: 3 points. Total scores are categorized as follows: 0–4 (none), 5–9 (mild), 10–14 (moderate), 15–19 (moderately severe), and 20–27 (severe) [27]. GAD-7 was used to assess anxiety disorders, with scores ranging from 0 to 3 based on frequency: not at all, several days, more than half the days, and nearly every day. Total scores were categorized as follows: 0–4 (none), 5–9 (mild), 10–14 (moderate), 15–21 (severe). The PHQ-15 consists of 15 somatic symptoms (stomach ache; back pain; pain in arms, legs, or joints; menstrual cramps or other problems with periods; headaches; chest pain; dizziness; fainting spells; feeling the heart pound or race; shortness of breath; pain or problems during sexual intercourse; constipation, loose bowels, or diarrhea; nausea, gas, or indigestion; feeling tired or having low energy; and trouble sleeping). Each symptom is rated from 0 (not bothered at all) to 2 (bothered a lot). Cut-off points of 5, 10, and 15 represent low, medium, and high levels of somatic symptom severity, respectively [23,26]. To confirm the applicability of these tools in our study population, we calculated Cronbach’s alpha reliability coefficients using data from the first 20 patients. All scales (OBC, GCPS 2.0, JFLS-8, PHQ-9, GAD-7, PHQ-15) achieved values > 0.7, indicating acceptable internal consistency [28].

Pain intensity was assessed using the Visual Analog Scale (VAS), a 100-millimeter horizontal line anchored at one end by “no pain” and at the other by “worst pain”. Participants indicated their perceived pain intensity by marking a point on the line. The distance, in millimeters, between the left anchor and the marked point was then measured [29].

### 2.5. Data Collection and Bias

**Figure 1:**
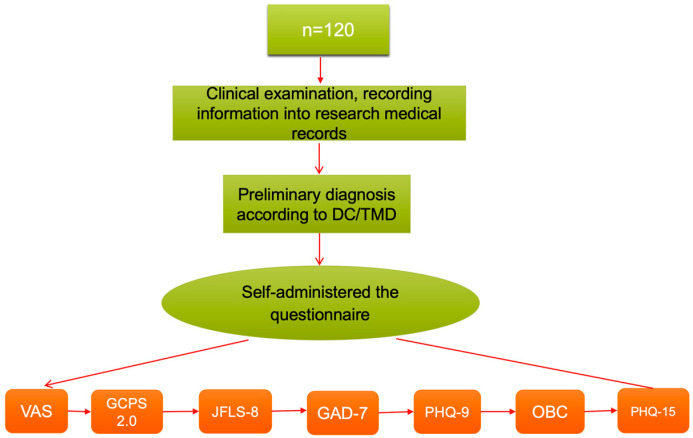
Cross-sectional study design. Abbreviations: VAS = Visual Analog Scale; GCPS 2.0 = Graded Chronic Pain Scale; JFLS-8 = Jaw Functional Limitation Scale 8; GAD-7 = General Anxiety Disorder 7; PHQ-9 = Patient Health Questionnaire 9; OBC = Oral Behavior Checklist; PHQ-15 = Patient Health Questionnaire 15.

All standardized examinations and participant guidance were conducted by the principal investigator under the close supervision of the academic advisor to minimize bias during data collection. To reduce recall bias, specific, short-term reference periods were employed in the questionnaires: 14 days for the PHQ-9 and GAD-7, and one month for the JFLS-8 and OBC questionnaires. All questionnaires were administered in a consultation room under the supervision of the principal investigator. The instruments were completed in the following order to minimize fatigue effects: VAS → CPS 2.0 → JFLS-8 → GAD-7 → PHQ-9 → OBC → PHQ-15. Figure 1 illustrates this sequence.

### 2.6. Statistical Analysis

The data were entered and analyzed by SPSS 20.0 software. Post hoc power analysis was conducted to evaluate the adequacy of the sample size. With 120 patients, the study achieved over 80% power to detect medium effect sizes (Cohen’s d = 0.50, α = 0.05). Associations between categorical variables were assessed using Chi-square and Fisher’s exact tests. Spearman’s correlation was used to explore the relationships between GAD-7, PHQ-9, JFLS-8, VAS, and OBC scores. In addition, ordinal logistic regression was applied to identify independent predictors of oral behaviors (as ordinal variables scored 0–4 from the OBC), adjusting for age, sex, pain intensity (VAS), and pain duration. For analyses examining the association between OBC and anxiety (GAD-7), models were further adjusted for depression (PHQ-9), and vice versa. Statistical significance was set at the 0.05 level.

## 3. Results

### 3.1. Demographic Characteristics

One hundred twenty patients with TMD were included, of whom 85 (70.8%) were female and 35 (29.2%) were male. The mean age was 30.92 ± 12.16 years. The mean OBC score was 18.7 ± 9.1; 43.3% had scores within the normal range (0–16), 35.8% moderate (17–24), and 20.8% high (≥25).

### 3.2. Correlation and Univariate Analysis

Correlation analysis showed that pain intensity (VAS) was strongly associated with chronic pain severity (GCPS) and moderately correlated with depression (PHQ-9) and somatic symptoms (PHQ-15). GCPS was also correlated with anxiety (GAD-7) and depression (PHQ-9), while anxiety and depression were strongly interrelated (r = 0.650, *p* < 0.001) (Supplementary Table S2).

In univariate comparisons, GCPS was significantly associated with OBC categories (*p* = 0.032; Table 1). Age, gender, mandibular function, and pain duration were not significantly associated with OBC scores (all *p* > 0.05). (Tables S1 and S3).

Differences in jaw pressure during sleep were observed among pain disorder subgroups (*p* = 0.044; Table 2).

### 3.3. Associations with Anxiety and Depression

The prevalence of mild, moderate, and severe anxiety was 25.0%, 15.8%, and 4.2%, respectively. The prevalence of mild depression was 30.0%, moderate depression 5.8%, and moderately severe to severe depression 2.5%. Higher anxiety levels were significantly associated with specific oral behaviors, including nocturnal bruxism, diurnal bruxism, jaw pressure during sleep, and gum chewing (*p* = 0.007, *p* = 0.041, *p* = 0.011, and *p* = 0.014, respectively; Table 2). Depression levels did not show a significant association with oral behaviors, although a tendency was observed in individuals with severe depression (Table S3).

### 3.4. Multivariate Analysis

Ordinal logistic regression confirmed that anxiety (GAD-7) was independently associated with nocturnal bruxism (OR = 2.95, 95% CI: 1.30–6.67, *p* = 0.010). After adjusting for age, sex, depression, and duration, each 1-point increase in anxiety score was associated with nearly 3-fold higher odds of reporting more frequent nocturnal bruxism. Other oral behaviors were not significantly related to anxiety. After adjusting for age, se anxiety (GAD-7), pain intensity (VAS), and pain duration, depression (PHQ-9) showed no independent association with any oral behaviors (Table 3).

In the multivariable linear regression model, only pain intensity (VAS) was independently associated with overall OBC scores (Coef = 1.829, 95% CI: 0.51–3.15, *p* = 0.007) after controlling for psychosocial and functional variables. Anxiety, depression, jaw functional limitation, and somatic symptoms were not significant predictors (all *p* > 0.05) (Table 4).

Supplementary analyses supported these findings, with age showing a negative association with oral behavior categories, and no significant effects observed for pain subgroups or pain duration (Supplementary Tables S3–S6).

## 4. Discussion

This study demonstrates that the associations between temporomandibular disorders (TMDs), oral behaviors, and psychosocial factors differed between univariate and multivariable analyses. Such discrepancies indicate that relying solely on unadjusted findings may lead to misleading clinical interpretations and emphasize the necessity of multivariable approaches to identify clinically meaningful risk factors.

Gender and mandibular function were not significantly associated with oral behaviors, which is consistent with previous studies reporting no clear relationship between oral parafunctional habits and sex differences [17]. However, our results contradict previous studies suggesting that females exhibit higher rates of oral habits and parafunctional behaviors than males. Parafunctional activities might contribute to differences in the prevalence of signs and symptoms of TMDs between the two genders [30]. Moreover, while age was not associated with OBC levels in the univariate analysis, multivariable adjustment revealed an inverse relationship, indicating that older patients reported fewer parafunctional habits. This finding underscores the influence of confounding factors, particularly pain intensity, and supports recent evidence that bruxism and oral behaviors tend to decline with advancing age [31].

### 4.1. Oral Behaviors and TMD Subgroups, Duration of Pain

Oral behaviors have been implicated as a contributing factor to the development of TMD signs and symptoms. Pain is the most common and bothersome symptom for patients seeking treatment. Consistent with previous studies, our multivariable analysis showed that patients with TMD-related pain were more likely to exhibit parafunctional oral behaviors compared to those with lower pain scores [12]. However, our study showed no significant difference in the duration of pain between different oral behavior groups (Table S6). Giorgio Iodice [32] also demonstrated a significant association between oral behaviors and TMD pain. There was a positive association between specific oral behaviors and TMD symptoms. Individuals with a higher frequency of specific oral behaviors are more likely to suffer from more TMD symptoms [18]. Nevertheless, some studies have concluded that there is no correlation between oral behaviors and TMD pain [33]. The discrepancies in findings across studies may be attributed to the use of different diagnostic criteria for TMDs and pain assessment scales.

In univariate analysis, our findings indicated that sleep posture, specifically placing pressure on the jaw, differed significantly among TMD subtypes, with the combined pain group (Myalgia + Arthralgia) exhibiting the highest prevalence. However, multivariable adjustment (controlling for age, gender, GAD-7, PHQ-9, pain intensity, and pain duration) revealed no independent association between specific oral behaviors and TMD pain subgroups (Tables S3 and S5). This suggests that confounding factors may drive the observed group differences, rather than subtype-specific behavior patterns. Our results are consistent with recent research showing that oral behaviors in isolation do not predict specific TMD subgroups when controlling for key variables [34]. Previous research has shown differential associations between oral behaviors and various TMD subgroups in female patients [14]. However, the subgroups considered by these authors included pain-related, intra-articular, combined pain-related intra-articular, and non-TMD groups, which differ from our study. Kella found no association between specific oral behaviors and primary pain diagnosis (muscle or joint pain) based on TMD diagnosis. Oral behaviors are differentially associated with various TMD subgroups in female adults [15].

### 4.2. Oral Behaviors and Anxiety Disorders, Depression

Anxiety and depression are frequently observed in patients with chronic orofacial pain, with higher prevalence rates compared to pain-free controls in studies with control groups, and are closely linked to pain severity [16]. TMD patients exhibit a higher frequency of oral behaviors compared to healthy individuals. This is particularly evident in TMD patients with pain and psychosocial problems [14]. At the univariate level, four oral behaviors: clenching or grinding teeth during sleep, sleeping in a position that places pressure on the jaw, grinding teeth during waking hours, and gum chewing, were significantly associated with GAD-7 anxiety levels. Yet, in the adjusted model, only nocturnal bruxism (clenching or grinding teeth when asleep) remained independently related to anxiety. Many authors have argued that the persistence of sleep bruxism as an independent factor associated with anxiety is consistent with previous evidence showing that sleep bruxism is closely linked to psychological stress and anxiety-related arousal mechanisms [35]. Despite established associations between parafunctional oral behaviors and TMD pain, the literature lacks particular behaviors that are associated with anxiety levels [12,18]. Or have only explored the correlation between oral behaviors and the presence/absence of anxiety or depression, and have limited their scope to female subjects [14]. Certain oral behaviors showed a statistically significant association with chronic pain, including sleep posture with jaw pressure [15]. As anxiety levels increased, so too did the frequency of these behaviors. We observed that individuals with higher levels of anxiety were more likely to engage in tooth grinding or clenching during sleep. Anxiety may be a contributing factor in the development and maintenance of harmful oral behaviors.

However, we found no clear evidence of a relationship between depression levels (PHQ-9) and the assessed oral behaviors. While some behaviors showed a slight increase in the severely depressed group, this difference was insufficient to conclude a causal relationship. Thus, the association between pain conditions and anxiety disorders is often stronger than that between pain conditions and depression. These findings add to the growing body of evidence suggesting that anxiety disorders should receive greater attention in the context of pain [36]. Our findings did not support a significant positive correlation between depression and oral behaviors. This is contrary to the findings of Lili Xu, who reported a significant positive correlation between oral behaviors and both anxiety and depression [17]. Khawaja reported that only physical and depressive symptoms were significant predictors of waking-state oral parafunctional behaviors [37]. Participants with a higher frequency of oral behaviors reported significantly higher anxiety and stress compared to participants with a lower frequency of oral behaviors [38]. This suggests that individuals with more severe anxiety and depression may exhibit more parafunctional behaviors, creating a vicious cycle of risk factors that perpetuate and exacerbate the condition [17].

Anxiety and depression present distinct neurobiological and psychological profiles, potentially explaining their differential associations with oral behaviors. Anxiety disorders are characterized by hyperactivity in the amygdala, a brain region crucial for processing fear and emotional responses. This increased activity can lead to heightened vigilance and repetitive behaviors, such as oral habits (e.g., nail-biting, lip-chewing), which may serve as coping mechanisms to mitigate tension [39]. Conversely, depression is often associated with hypoactivity in the prefrontal cortex and disruptions in neurotransmitter systems, including serotonin and dopamine pathways. These alterations can result in diminished motivation and psychomotor retardation, making the emergence of repetitive oral behaviors less prevalent in depressed individuals [40].

### 4.3. Oral Behaviors and Chronic Pain

According to the American Pain Society Pain Taxonomy, chronic TMD is the persistence of pain for over 3 months, based on a reasonable period for biological healing response to any tissue damage [41]. Findings regarding the relationship between oral behaviors and chronic pain severity (GCPS) were not entirely consistent across analytic approaches. In the univariate analysis (Table 1), GCPS emerged as the only factor significantly associated with oral behaviors, suggesting that higher pain severity was linked to changes in oral parafunctions. A subsequent correlation analysis (Table S2) showed a positive trend between chronic pain scores and OBC, although this association did not reach statistical significance. In contrast, supplementary univariate testing (Table S4) did not demonstrate any significant relationship between GCPS and OBC levels. These discrepancies may be explained by differences in the analytic methods, sample stratification. Multivariable models remain essential to disentangle true independent effects from spurious or method-related findings. Other authors have concluded that a notable correlation was observed between levels of OBC and chronic pain [15,42].

Several oral behaviors were significantly associated with chronic pain, including “sleep in a position that puts pressure on the jaw,” “hold, tighten, or tense muscles without clenching or bringing teeth together,” “hold or just jaw forward or to the side,” “press tongue forcibly against teeth,” “place tongue between teeth,” “hold the jaw in a rigid or tense position,” and “yawning.” The multivariate regression analysis identified “place tongue forcibly against teeth” as the strongest predictor of chronic pain [15]. As parafunctional habits increase, the masticatory muscles and temporomandibular joint are forced to work when they should be resting, thereby increasing the load on the temporomandibular joint, prolonging pain symptoms, and leading to chronic pain [1].

The predominant method for assessing the relationship between oral behaviors (including bruxism and other behaviors) and TMDs has been through self-reported questionnaires. These studies have consistently shown a significant association with bruxism [11,43,44]. However, studies using objective measures, such as tooth wear or electromyography measures, have yielded conflicting results, with some finding no association [10,45]. Our findings contrasted with previous studies that reported no association between oral behaviors and TMD pain intensity [15,33]. In line with the work of Lili Xu [17], however, we observed a significant positive correlation between pain intensity and OBC scores. Individuals with pain-related TMD had statistically significantly higher OBC scores compared to normal or non-painful individuals [37]. Participants with the highest frequency of oral behaviors experienced clinically significant more TMD pain than those with the lowest frequency [38]. Other studies have identified specific oral behaviors associated with pain intensity, such as holding a telephone between the head and shoulders, which was significantly associated with mild to moderate pain [15].

Pain intensity emerged as the only independent predictor of overall oral behavior scores in the multivariable analysis, while anxiety was specifically associated with nocturnal bruxism and depression showed no independent effect. These findings suggest that pain burden plays a central role in shaping oral parafunctional behaviors, with anxiety exerting a more selective influence [46]. From a clinical perspective, this underscores the importance of integrating pain assessment with evaluation of psychosocial status when managing TMD patients. Although psychological distress may not independently predict all oral behaviors, it remains an important modifier of patient experience and should be considered in individualized treatment planning [36,47]. Ultimately, effective management of TMD requires a comprehensive approach that addresses not only pain and oral behaviors but also the broader psychosocial context of each patient [46].

### 4.4. Limitations and Future Directions

This study utilized a reliable self-reported questionnaire provided by DC/TMD, yet it has inherent limitations. Self-reported measures may have introduced reporting errors. Although multivariable analyses allowed adjustment for confounders, the cross-sectional design prevents causal inference, and the lack of a control group limits full control of bias. Additionally, as a cross-sectional study with a limited sample size, it may not be generalizable to the broader population. Future research should employ larger sample sizes and include control groups. Standardized tools should be utilized to identify individuals with bruxism, particularly sleep bruxism, enabling a more precise assessment of the frequency of oral behaviors.

## 5. Conclusions

Our findings revealed that, in univariate analyses, several psychosocial factors appeared to be associated with oral behaviors. However, after multivariable adjustment, pain intensity remained the only independent predictor of overall OBC scores, while anxiety was specifically related to nocturnal bruxism, and depression showed no independent effect.

**Recommendation:** A comprehensive treatment approach for TMD patients should include patient education and behavioral modification, with a particular focus on reducing parafunctional oral behaviors to minimize the risk of relapse and prolong pain relief.

## Figures and Tables

**Table 1:** Distribution of oral behaviors according to chronic pain score (GCPS). **Abbreviations: OBC** = Oral Behavior Checklist; **GCPS** = Graded Chronic Pain Scale.

GCPS (*n*, %)	OBC 0–16	OBC 17–24	OBC ≥ 25	*p*-Value
Grade 0	2 (100)	0 (0)	0 (0)	0.032 **
Grade I	13 (40.6)	12 (37.5)	7 (21.9)	
Grade II	35 (48.6)	27 (37.5)	10 (13.9)	
Grade III	2 (18.2)	3 (27.3)	6 (54.5)	
Grade IV	0 (0)	1 (33.3)	2 (66.7)	
120 (100.0)	52 (43.3)	43 (35.8)	25 (20.8)	

* Chi-square test, ** Fisher’s exact test. Note: Bold indicates statistically significant results (*p* < 0.05).

**Table 2:** Associations of oral behaviors with pain disorder subgroup and anxiety (GAD-7). **Abbreviations: GAD-7** = General Anxiety Disorder 7.

Oral Behavior	PAIN DISORDER SUBGROUPS					
	**No Pain (*n*, %)**	**Myalgia (*n*, %)**	**Arthralgia (*n*, %)**	**Combined Pain (*n*, %)**	**Total (*n*, %)**	***p*-Value**
**Sleep in a position that puts pressure on the jaw**	2 (2.0)	15 (15.2)	8 (8.1)	74 (74.7)	99 (82.5)	0.044 **
	**GAD-7**					
	**None (*n*, %)**	**Mild (*n*, %)**	**Moderate (*n*, %)**	**Severe (*n*, %)**	**Total (*n*, %)**	***p*-value**
**Clench or grind teeth when asleep**	25 (44.7)	13 (23.2)	13 (23.2)	5 (8.9)	56 (46.7)	0.007 *
**Sleep in a position that puts pressure on the jaw**	51 (51.5)	29 (29.3)	14 (14.1)	5 (5.1)	99 (82.5)	0.041 *
**Grind teeth together during walking hours**	16 (37.2)	14 (32.6)	9 (20.9)	4 (9.3)	43 (35.8)	0.011 **
**Use chewing gum**	26 (54.2)	13 (27.1)	4 (8.3)	5 (10.4)	48 (40)	0.014 **

* Chi-square test, ** Fisher’s exact test. Note: Bold indicates statistically significant results (*p* < 0.05).

**Table 3:** Association between anxiety and depression levels and oral behaviors (ordinal logistic regression). **Abbreviations: GAD-7** = General Anxiety Disorder 7; **PHQ-9** = Patient Health Questionnaire 9.

Oral Behaviors	GAD-7			PHQ-9		
	Coef	OR (95% CI)	*p*-Value	Coef	OR (95% CI)	*p*-Value
**Clench or grind teeth when asleep**	1.081	2.95 (1.30–6.67)	**0.01**	−0.106	0.90 (0.37–2.19)	0.815
**Sleep in a position that puts pressure on the jaw**	1.032	2.81 (0.81–9.72)	0.104	0.229	1.26 (0.35–4.50)	0.724
**Grind teeth together during walking hours**	0.75	2.12 (0.91–4.94)	0.083	0.111	1.12 (0.45–2.77)	0.81
**Use chewing gum**	0.259	1.30 (0.55–3.06)	0.555	0.658	1.93 (0.75–4.96)	0.171
**Chew food on one side only**	0.184	1.20 (0.30–4.82)	0.795	−0.293	0.75 (0.18–3.11)	0.687
**Sustained talking**	0.041	1.04 (0.44–2.48)	0.926	0.407	1.50 (0.60–3.78)	0.387
**Yawning**	−0.173	0.84 (0.28–2.52)	0.758	−0.13	0.88 (0.27–2.85)	0.828

Note: *p* < 0.05 was considered statistically significant.

**Table 4:** Multivariable linear regression of factors associated with oral behavior scores.

Variable	Coef (95% CI)	*p*-Value
GAD-7	0.454 (−0.03–0.94)	0.067
PHQ-9	0.243 (−0.24–0.72)	0.317
JFLS-8	−0.044 (−0.20–0.11)	0.57
PHQ-15	0.011 (−0.49–0.51)	0.965
VAS	1.829 (0.51–3.15)	**0.007**

Note: Results from multivariable linear regression adjusted for age, gender, depression (PHQ-9), anxiety (GAD-7), jaw functional limitation (JFLS-8), somatic symptoms (PHQ-15), pain intensity (VAS), and pain duration. Bold indicates statistically significant results (*p* < 0.05).

## Data Availability

All data generated or analyzed during this study are included in this published article.

## Supplementary Materials

The following supporting information can be downloaded at: https://www.mdpi.com/article/10.3390/dj13100480/s1, Table S1: Distribution of oral behaviors according to gender, age, mandibular function, and chronic pain score; Table S2: Correlation matrix of VAS, GCPS, JFLS-8, GAD-7, PHQ-9, PHQ-15, duration of pain, and OBC; Table S3: Association between oral behavior and duration of pain, pain disorder subgroups, and psychological factors (GAD-7, PHQ-9); Table S4: Factors associated with oral behavior categories; Table S5: Association between oral behaviors and pain subgroups (multinomial logistic regression); Table S6: Association between duration of pain and oral behaviors.

## Abbreviations

TMDs = Temporomandibular joint disorders; DC/TMD = Diagnostic criteria for temporomandibular disorders; GAD-7 = General anxiety disorder 7; GCPS = Graded chronic pain scale; JFLS-8 = Jaw functional limitation scale 8; OBC = Oral behavior checklist; PHQ-9 = Patient health questionnaire 9; PHQ-15 = Patient health questionnaire 15; VAS = Visual analog scale.
