# Supplementary material for: Assessment of Common Oral Behaviors in Patients with Temporomandibular Joint Disorders and Their Relationship to Psychosocial Factors
Source: Dent J (Basel). 2025 Oct 20;13(10):480. doi: 10.3390/dj13100480 (PMC12564427; doi:10.3390/dj13100480)
Supplement: Supplementary file 1 [file dentistry-13-00480-s001.zip › dentistry-3830492-supplementary.pdf]

Supplementary Tables

Manuscript ID: dentistry-3830492

Title: Assessment of Common Oral Behaviors in Patients with Temporomandibular Joint Disorders and their Relationship to Psychosocial Factors.

Authors: Nguyen Ngoc Hoa, Hoang Viet Hai, Tran > Thai Binh, To Thanh Dong, Tran Thi Minh Quyen, Toan Do *

Table S1. Distribution of oral behaviors according to gender, age, mandibular function, and chronic pain score. **Abbreviations: OBC** = Oral Behavior Checklist; **JFLS-8** = Jaw Functional Limitation Scale 8; **GCPS** = Graded Chronic Pain Scale.

Parameter.	OBC	0-16	17-24	≥25
	Male	19 (54.3)	7 (20)	9 (25.7)
**Gender (n,%)**	Female	33 (38.8)	36 (42.4)	16 (18.8)
	P-value	.068*		
	<20	7 (36.8)	6 (31.6)	6 (31.6)
	20-29	15 (31.9)	11 (23.4)	6 (18.8)
**Age (n,%)**	30-39	16 (50)	10 (31.3)	6 (18.8)
	>=40	14 (63.6)	6 (27.3)	2 (9.1)
	P-value	.203**		
	<5	2 (100)	0 (0)	0 (0)
**JFLS-8 (n,%)**	>=5	50 (42.4)	43 (36.4)	25 (21.2)
	P-value	.687**		
	Grade 0	2 (100)	0 (0)	0 (0)
	Grade I	13 (40.6)	12 (37.5)	7 (21.9)
	Grade II	35 (48.6)	27 (37.5)	10 (13.9)
**GCPS (n,%)**	Grade III	2 (18.2)	3 (27.3)	6 (54.5)
	Grade IV	0 (0)	1 (33.3)	2 (66.7)
	P-value	**.032****		
**Total (n,%)**	120	52 (43.3)	43 (35.8)	25 (20.8)

* Chi-square test, **Fisher's exact test.

Table S2. Correlation matrix of VAS, GCPS, JFLS-8, GAD-7, PHQ-9, PHQ-15, duration of pain, and OBC. **Abbreviations: VAS** = Visual Analog Scale; **GCPS** = Graded Chronic Pain Scale; **JFLS-8** = Jaw Functional Limitation Scale 8; **GAD-7** = General Anxiety Disorder 7; **PHQ-9** = Patient Health Questionnaire 9; **PHQ-15** = Patient Health Questionnaire 15; **OBC** = Oral Behavior Checklist.

Spearman's coefficient	Duration of pain	GCPS	GAD-7	PHQ-9	OBC	PHQ-15	
**VAS**	0.177	0.582**	0.111	0.291**	0.167	0.286**	0.7
**Duration of pain**		0.216*	0.181*	0.174	0.104	0.198*	0.6
**GCPS**			0.304**	0.324**	0.170	0.304**	0.5
**GAD-7**				0.650**	0.084	0.348**	0.4

**PHQ-9**	0.164	0.414**		0.3
**JFLS-8**	0.071	0.317**		0.2
**OBC**		0.037		0.1

Spearman's coefficient test; *Correlation is significant at the .05 level; ** Correlation is significant at the .01 level.

Table S3. Association between oral behavior and duration of pain, pain disorder subgroups, and psychological factors (GAD-7, PHQ-9). **Abbreviations: GAD-7** = General Anxiety Disorder 7; **PHQ-9** = Patient Health Questionnaire 9.

Parameter	Clench or grind teeth when asleep	Sleep in a position that puts pressure on the jaw	Grind teeth together during walking hours	Use chewing gum	Chew food on one side only	Sustained talking	Yawning	Other oral behaviors
**DURATION OF PAIN**								
**None**	2 (66.7)	2 (66.7)	1 (33.3)	2 (66.7)	2 (66.7)	2 (66.7)	2 (66.7)	3 (100)
**Acute <3 months (n,%)**	43 (46.7)	76 (82.6)	29 (31.5)	37 (40.2)	83 (90.2)	59 (64.1)	77 (83.7)	85 (92.4)
**Chronic ≥3 months (n,%)**	11 (44)	21 (84)	13 (52)	9 (36)	23 (92)	21 (84)	21 (84)	24 (96)
**Total**	56 (46.7)	99 (82.5)	43 (35.8)	48 (40)	108 (90)	82 (68.3)	100 (83.3)	112 (93.3)
**P-value**	.738**	.657**	.138**	.620**	0.366**	.127**	.636**	1.000**
**PAIN DISORDER SUBGROUPS**								
**No pain (n,%)**	2 (3.6)	2 (2.0)	1 (2.3)	2 (4.2)	2 (1.9)	2 (2.4)	2 (2.0)	3 (2.7)
**Myalgia (n,%)**	10 (17.9)	15 (15.2)	9 (20.9)	7 (14.6)	20 (18.5)	14 (17.1)	18 (18.0)	20 (17.8)
**Arthralgia (n,%)**	5 (8.9)	8 (8.1)	2 (4.7)	3 (6.2)	7 (6.5)	5 (6.1)	5 (5.0)	7 (6.3)
**Combined pain (n,%)**	39 (69.6)	74 (74.7)	31 (72.1)	36 (75.0)	79 (73.1)	61 (74.4)	75 (75.0)	82 (73.2)
**Total (n,%)**	56 (46.7)	99 (82.5)	43 (35.8)	48 (40)	108 (90)	82 (68.3)	100 (83.3)	112 (93.3)
**P-value**	.701**	**.044****	.909**	.608**	.256**	.774**	.141**	.298**
**GAD-7**								
**None (n,%)**	25 (44.7)	51 (51.5)	16 (37.2)	26 (54.2)	59 (54.6)	43 (52.4)	56 (56.0)	61 (54.5)
**Mild (n,%)**	13 (23.2)	29 (29.3)	14 (32.6)	13 (27.1)	28 (25.9)	22 (26.8)	26 (26.0)	29 (25.9)
**Moderate (n,%)**	13 (23.2)	14 (14.1)	9 (20.9)	4 (8.3)	17 (15.8)	12 (14.7)	13 (13.0)	17 (15.2)
**Severe (n,%)**	5 (8.9)	5 (5.1)	4 (9.3)	5 (10.4)	4 (3.7)	5 (6.1)	5 (5.0)	5 (4.4)
**Total (n,%)**	56 (46.7)	99 (82.5)	43 (35.8)	48 (40)	108 (90)	82 (68.3)	100 (83.3)	112 (93.3)
**P-value**	**.007***	**.041***	**.011****	**.014****	.635**	.429*	.281*	.72*
**PHQ-9**								
**None (n, %)**	29 (51.8)	56 (56.6)	21 (48.8)	27 (56.3)	65 (60.2)	48 (58.5)	59 (59.0)	67 (59.8)
**Mild (n, %)**	19 (33.9)	31 (31.3)	14 (32.6)	13 (27.1)	33 (30.6)	22 (26.8)	29 (29.0)	33 (29.5)
**Moderate (n, %)**	4 (7.1)	6 (6.1)	3 (7.0)	3 (6.2)	5 (4.6)	6 (7.3)	6 (6.0)	6 (5.3)
**Moderately severe (n, %)**	1 (1.8)	3 (3.0)	2 (4.7)	2 (4.2)	2 (1.8)	3 (3.7)	3 (3.0)	3 (2.7)
**Severe (n, %)**	3 (5.4)	3 (3.0)	3 (6.9)	3 (6.2)	3 (2.8)	3 (3.7)	3 (3.0)	3 (2.7)
**Total (n, %)**	56 (46.7)	99 (82.5)	43 (35.8)	48 (40)	108 (90)	82 (68.3)	100 (83.3)	112 (93.3)

**P-value**	.251**	.933**	.071**	.229**	.221**	.453**	.973**	.646**

*Chi-square test, **Fisher's exact test.

Table S4. Factors associated with oral behavior categories. **Abbreviations: GCPS** = Graded Chronic Pain Scale

Variable	OR (95% CI)	p-value
**Age**	0.95 (0.92–0.98)	**0.002***
**Sex**	1.04 (0.46–2.35)	0.928
**GCPS**	1.17 (0.59–2.30)	0.659

Note: Results from multivariable ordinal logistic regression adjusted for age, sex, anxiety (GAD-7), depression (PHQ-9), pain intensity (VAS), and pain duration. p < 0.05 was considered statistically significant.

Table S5. Association between oral behaviors and pain subgroups (multinomial logistic regression)

Behavior (predictor)	Comparison	Coef	OR (95% CI)	p-value
**Clench or grind teeth when asleep**	Myalgia vs Combined	0.763	2.14 (0.34–13.43)	0.415
**Clench or grind teeth when asleep**	Arthralgia vs Combined	0.076	1.08 (0.39–2.95)	0.882
**Sleep in a position that puts pressure on the jaw**	Myalgia vs Combined	17.361	NE	0.994
**Sleep in a position that puts pressure on the jaw**	Arthralgia vs Combined	1.223	3.40 (0.96–12.03)	0.058
**Grind teeth together during walking hours**	Myalgia vs Combined	-1.915	0.15 (0.01–1.63)	0.118
**Grind teeth together during walking hours**	Arthralgia vs Combined	-0.544	0.58 (0.20–1.64)	0.306
**Use chewing gum**	Myalgia vs Combined	0.248	1.28 (0.20–8.22)	0.794
**Use chewing gum**	Arthralgia vs Combined	0.599	1.82 (0.60–5.54)	0.292
**Chew food on one side only**	Myalgia vs Combined	-0.186	0.83 (0.06–11.04)	0.888
**Chew food on one side only**	Arthralgia vs Combined	0.69	1.99 (0.42–9.52)	0.387
**Sustained talking**	Myalgia vs Combined	-0.539	0.58 (0.09–3.76)	0.571
**Sustained talking**	Arthralgia vs Combined	0.396	1.49 (0.52–4.24)	0.459

**Yawning**	Myalgia Combined	vs	-0.86	0.42 (0.05–3.32)	0.413
**Yawning**	Arthralgia Combined	vs	0.5	1.65 (0.43–6.34)	0.466

Note: Multinomial logistic regression model with Combined pain as the reference category, adjusted for age, gender, GAD-7, PHQ-9, pain intensity (VAS), and pain duration. $p < 0.05$ was considered statistically significant.

Table S6. Association between duration of pain and oral behaviors.

Oral Behaviors	Coef	OR (95% CI)	p-value
**Clench or grind teeth when asleep**	-0.183	0.83 (0.33–2.11)	0.7
**Sleep in a position that puts pressure on the jaw**	-0.036	0.97 (0.28–3.36)	0.956
**Grind teeth together during walking hours**	0.776	2.17 (0.82–5.75)	0.118
**Use chewing gum**	-0.062	0.94 (0.35–2.50)	0.902
**Chew food on one side only**	0.561	1.75 (0.40–7.76)	0.46
**Sustained talking**	0.825	2.28 (0.79–6.58)	0.127
**Yawning**	0.162	1.18 (0.34–4.07)	0.799

Note: Results from ordinal logistic regression adjusted for age, gender, anxiety (GAD-7), depression (PHQ-9), and pain intensity (VAS). $p < 0.05$ was considered statistically significant.
